# Impact of minimum bid requirement of Japan's electricity market on virtual power plant's profit

**DOI:** 10.1016/j.heliyon.2025.e42067

**Published:** 2025-01-16

**Authors:** Reza Nadimi, Masahito Takahashi, Koji Tokimatsu, Mika Goto

**Affiliations:** a Department of Innovation Science, School of Environment and Society, Tokyo Institute of Technology, 3-3-6, Shibaura, Minato-ku, Tokyo, 108-0023, Japan; b Central Research Institute of Electric Power Industry, Ōtemachi, Tokyo, 100-8126, Japan; c Department of Transdisciplinary Science and Engineering, School of Environment and Society, Tokyo Institute of Technology, 4259 Nagatsuta-cho, Midori-ku, Yokohama, 226-8503, Japan

**Keywords:** Virtual power plant, Simulation-based optimization, Short-term power market, Power market design options

## Abstract

In the context of small-scale virtual power plants (VPPs), the minimum power requirement (MPR) poses a significant challenge for market participation due to the inherent volatility and uncertainty associated with renewable energy sources. To mitigate this, a VPP system can potentially enhance its profitability by utilizing an Internal MPR, which is a value derived from market prices and historical data, including actual and forecasted renewable power generation, and is typically lower than the market MPR. This study investigates several market strategies, designated as Plans II, III, and V, under hypothetical conditions, and evaluates their impact on the profitability of VPPs relative to existing strategies (Plans I and IV) within the Japan Electric Power eXchange (JEPX) market. Currently, the MPR for day-ahead (DA) and intraday (ID) markets is set at 0.1 MW per settlement period. This research involves the simulation of all five plans across 11 small-scale VPP systems, with capacities ranging from 0.05 MW to 1 MW. The simulation results indicate that reducing the market MPR to 0.04 MW for both DA and ID markets results in increased profitability for VPPs. The findings of this study suggest potential policy implications for Japan's power market, emphasizing the benefits of adjusting MPR thresholds to better accommodate the participation of small-scale VPPs and enhance overall market efficiency.

## Highlights

• Internal minimum power requirement (MPR) should be calculated by virtual power plant (VPP) owners.
• The internal MPR increase VPP profit.
• Joint probability distribution was proposed to calculate the internal MPR.

**Table:** Nomenclature

Majority of variables' format: $A_{C}^{B,D}$ A refers to Demand, Generation, Supply, Price, Surplus, State of charge (SOC), Cost, Binary/Slack variables, B specifies source of power supply (technology), demand sources, market types, negative/positive slack variable C indicates settlement period, minimum or maximum capacity for a technology, and D represents additional information such as estimated or actual power supply, For example, $S_{t}^{grid,E}$: represent estimated grid supply power at settlement period *t*.		
**Input Variables**	**Description**	**Unit**
$P_{t}^{DA}$	Day-ahead power price at period *t*	$/kW
$P_{t}^{ID}$	Intra-day power price at period *t*	$/kW
$P_{t}^{Bal}$	Balance cost at period *t*	$/kW
$R_{t}^{su}$	Random number generated from standard uniform distribution at period *t*	–
$R_{t}^{h}$	Historical percentage of contract's number at period *t* (per maximum daily contract)	–
$S_{t}^{VPP,E}$	Estimated VPP power supply at period *t*	kW
$S_{t}^{VPP,Bid}$	VPP power supply based on bid data for DA (ID) market at period *t*	kW
$S_{t}^{VPP,A}$	Actual VPP power supply at period *t*	kW
$\lambda_{1,2,3,4,t}{,\alpha }_{1,2,3,4,t}$	Trading transition probability at period *t*	%
**Intermediate Variables**	**Description**	**Unit**
${SOC}_{t}^{Bat}$	Battery state of charge at period *t*	kW
**Decision variable**	**Description**	**Unit**
**Continuous**		
$S_{t}^{Bat,disch}$	Discharging power from battery at period *t*	kW
$S_{t}^{Bat,ch}$	Charging power into battery at period *t*	kW
$S_{t}^{grid,E}$	Estimated grid power supply at period *t*	kW
$S_{t}^{grid,E,ID}$	Estimated grid power supply in the ID market at period *t*	kW
$S_{t}^{grid,E,Bal}$	Estimated grid power supply in the Balance market at period *t*	kW
$S_{t}^{grid,A,ID}$	Actual grid power supply in the ID market at period *t*	kW
$S_{t}^{grid,A,Bal}$	Actual grid power supply in the Balance market at period *t*	kW
${Su}_{t}^{VPP,ID}$	VPP surplus power in the ID market at period *t*	kW
${Su}_{t}^{VPP,Bal}$	VPP surplus power in the Balance market at period *t*	kW
**Binary**		
$B^{DA}$	Binary variable for day-ahead market	
$B_{t}^{Bat,ch}$	Binary variable for battery charging at period *t*	{0,1}
$B_{t}^{Bat,disch}$	Binary variable for battery discharging at period *t*	{0,1}
**Slack variable**	**Description**	**Unit**
${Sl}_{t}^{Negative}$	Negative slack variable at period *t*	kW
${Sl}_{t}^{Positive}$	Positive slack variable at period *t*	kW
**Model Parameters**	**Description**	**Unit**
$S_{\max}^{Bat,disch}$	Maximum discharging power from battery at period *t*	kW
$C^{VPP}$	Operation cost of VPP	$/kW
$C^{Bat,var}$	Variable operation cost of Battery	$/kW/year
$C^{Bat,fixed}$	Fixed operation cost of Battery	$/kWh/year
${SOC}^{\max}$	Maximum SOC of battery (80 % of battery capacity)	kWh
${SOC}^{\min}$	Minimum SOC of battery (20 % of battery capacity)	kWh
$\eta_{ch}^{Bat}$	Battery charging efficiency	%
$\eta_{disch}^{Bat}$	Battery discharging efficiency	%
$\Phi_{DA}$	Minimum power requirement (MPR) for DA market	kW
$\Phi_{ID}$	MPR for ID market	kW
$\Phi_{DA,R}$	Internal MPR for DA market	kW
$\Phi_{ID,R}$	Internal MPR for ID market	kW
**Other symbols**	**Description**	
JEPX	Japan Electric Power Exchange	–
M_1_	Big number or Big-M (here M_1_ = initial battery capacity)	–

## 1 Introduction

The integration of small-scale Virtual Power Plants (VPPs) is pivotal in advancing the Japanese government's "Green Transformation" (GX) program, which aims to facilitate a low-carbon transition [1]. To effectively support this transition, it is crucial to carefully assess market-related policies, such as subsidies (e.g., feed-in tariffs, variable and fixed feed-in premiums), incentives, and the liberalization of power markets [2]. Power market liberalization creates a broader spectrum of pricing mechanisms and opportunities for both power consumers and generation companies, fostering a more flexible and competitive energy market.

An efficient and reliable electric power generation system is fundamental to market design, aiming to meet demand while addressing the preferences and constraints of market participants [3,4]. Thermal generators are traditionally the dominant power sources, characterized by large-scale capacities and high marginal costs. These reliable power stations can lower overall system costs by reducing the necessary reserve capacity [5]. Conversely, distributed energy resources (DERs) are capital-intensive but offer high efficiency and low variable costs [6,7]. Virtual power plants [8] aggregate generation from multiple DERs, which helps mitigate the intermittency of renewable sources, facilitates electricity trading [9], and optimizes DER preferences, thus enhancing VPP profitability [10].

Thermal power plants typically submit bids based on their dependable capacities, whereas VPPs' bids are closely tied to variable weather conditions. Market preferences are influenced by various operational factors, including dispatch intervals, gate closure times, and financial settlement periods, each of which affects market dynamics and participant behavior.

Dispatch Interval: This parameter represents the maximum time allowed for delivering power to a specific location^1^ and clearing operating reserves in real-time balancing markets.^2^ Empirical studies, such as those on the German balancing market, have shown that reducing the dispatch interval to 15 min can shift 17 % of balancing reserve from renewables to the intraday market [11]. Optimal dispatch durations, in conjunction with energy storage solutions^3^ [12], can improve the integration of renewables into the power system and reduce their volatility and uncertainty [13,14]. Dispatch intervals vary among independent system operators (ISOs), with intervals for New York ISO and California ISO being 5 and 15 min, respectively [15,16]. Different dispatch methods^4^ can minimize the total cost of the power system given a specific dispatch interval [17].

Gate Closure Time: In wholesale electricity markets, gate closure time is the final moment for submitting or revising bids [18]. Shorter gate closure intervals allow VPPs to submit bids with minimal forecasting errors, though this may influence costs [19]. For instance, simulations of the French power market revealed that deferring the balancing gate closure from 60 to 15 min increased operational costs by 0.08%–0.43 % [20].

Financial Settlement Period (Trading Interval or Bid Interval): The impact of settlement period granularity on short-term wholesale markets has been explored in various studies [21,22]. Shorter settlement intervals can affect short-term wholesale electricity prices and increase the market share of intermittent renewables. Studies of the Australian power market showed mixed results: one study found no immediate effect on spot prices but a long-term cumulative increase [23], while another indicated a reduction of up to 5 % in wholesale prices [24]. Analysis of the German-Austrian spot market revealed price declines of 11 %–28 % when the settlement period was reduced from 1 h to 15 min [25].

To our knowledge, the internal minimum power requirement (MPR), a critical preference for VPP owners—particularly for small-scale VPPs—has not been thoroughly investigated. The JEPX market mandates a minimum power requirement of 0.1 MW for participation in day-ahead (DA) and intraday (ID) markets. Small-scale VPPs must meet this market MPR, although their actual bids can be affected by the inherent uncertainty of renewable energy sources.

This study explores the effect of the Internal MPR on the profitability of small-scale VPPs. The Internal MPR is a value less than the market MPR which is calculated based on market prices and actual and forecasted renewable power generation. If the estimated power supply falls below the market MPR but meets or exceeds the Internal MPR, the VPP will base its bid on the market MPR. The disparity between the Internal MPR and the market MPR reflects the risk level a VPP assumes when submitting a bid exceeding its estimated power supply. This study calculates Internal MPR values for the DA and ID markets and simulates the power supply of small-scale VPPs, examining their interaction with the JEPX market.

Accordingly, three alternative plans (II, III, and V) are defined and compared with the existing JEPX market plans (I and IV) to assess their impact on VPP profitability. VPP profitability is influenced by the stochastic behavior associated with switching from upstream to downstream markets (e.g., DA to ID markets) in response to power surpluses and/or shortages. The JEPX, established in 2003, facilitates financial transactions and competition among market participants through forward (year/month/week), day-ahead (day), and intraday (hour/minute) markets [26]. Since the full liberalization of Japan's retail electricity market in April 2016, the JEPX spot market accounted for 30 % of national power demand in March 2022 [27]. The DA and ID markets are particularly attractive for short-term trading compared to the forward market, which has lower liquidity [28].

Paper Structure: Section 2 introduces the VPP system and the VPP simulation-based optimization model used to assess profitability under the defined plans within the JEPX market. Section 3 describes the results of the VPP simulation model in terms of the stated plans. Section 4 discusses the results in further details. Section 5 provides a summary of the main results and implication of the proposed idea in policy making.

## 2 Methodology

**Fig. 1:**
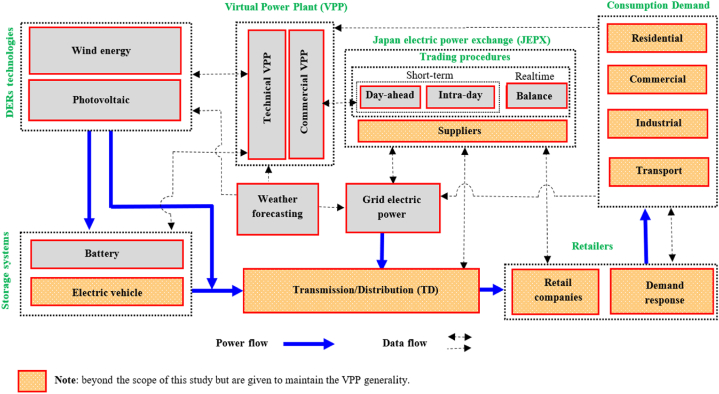
VPP framework and its interaction with power market and grid adopted from Ref. [10].

Fig. 1 illustrates the data and power flow within the centralized VPP system. The VPP itself is not the owner of the DERs, but it manages their power productions and sells them in the JEPX short-term and balance markets. Fig. 1 depicts the structure of the VPP, which comprises both commercial and technical components. The technical VPP is responsible for overseeing the real-time operation of DERs, including monitoring operational performance, managing associated costs, and ensuring effective utilization. Conversely, the commercial VPP engages with the power market by submitting bids, which are informed by power generation forecasts provided by the technical VPP [29]. The commercial VPP participates in short-term power markets using a consolidated power generation profile, which is derived by aggregating the daily generation profiles from the technical VPP. The profitability of the VPP is determined through its interactions with these power markets.

### 2.1 Descriptions of VPP simulation model

This study evaluates the VPP profitability under five different plans outlined in Table 1. Participants in the JEPX are required to meet a minimum power requirement of 0.1 MW with a half-hour settlement period in both the DA and ID markets [26]. Plan I and IV represent the actual conditions in the JEPX market, while the remaining plans assess potential changes in market profitability, primarily focusing on insights from small-scale VPP participants. The distinguishing factors among the plans include the market power requirement and market prices [25]. This study specifically analyzes three market strategies (Plans II, III, and V) under the Internal MPR condition, which is grounded in historical data rather than being purely theoretical. The primary objective of this analysis is to determine whether reducing the current JEPX market MPR could create opportunities for small-scale VPP participants to engage in the DA market, potentially yielding higher profits compared to the ID market.

**Table 1:** JEPX short-term market plans.

Market types	Day-ahead Market	Intraday Market	Note
Market requirements	Power requirement of market [MW]		
**Plan I**	$\Phi_{DA}=\Phi_{DA,R}=0.1$	$\Phi_{ID}=\Phi_{ID,R}=0.1$	Combination of DA markets and ID market
**Plan II**	$\Phi_{DA}=0.1\text{,}$ $\Phi_{DA,R}=\text{Internal}\;\text{MPR}$	$\Phi_{ID}=0.1\text{,}$ $\Phi_{ID,R}=\text{Internal}\;\text{MPR}$	Combination of DA markets and ID market
**Plan III**	$\Phi_{DA}=\Phi_{DA,R}=0.1$	$\Phi_{ID}=0.1\text{,}$ $\Phi_{ID,R}=\text{Internal}\;\text{MPR}$	Combination of DA markets and ID market
**Plan IV**		$\Phi_{ID}=\Phi_{ID,R}=0.1$	Just ID market
**Plan V**		$\Phi_{ID}=0.1\text{,}$ $\Phi_{ID,R}=\text{Internal}\;\text{MPR}$	Just ID market

Plan II and Plan I are differentiated primarily by their Internal MPR values and the short-term market prices. The selection between the DA and ID markets is influenced by the market price, which is incorporated into the objective function of the VPP system as described in Equations (8), (9), (10), (11). In Plan II, the Internal MPR serves as a "lower bound" for both DA and ID markets. Specifically, if the forecasted VPP supply for a given settlement period falls within the range [lower bound, 0.1 MW],^5^ the VPP system will submit a bid of 0.1 MW. This approach reflects the VPP system's willingness to take on the risk of submitting a bid that exceeds the forecasted power generation from DERs.

**Fig. 2:**
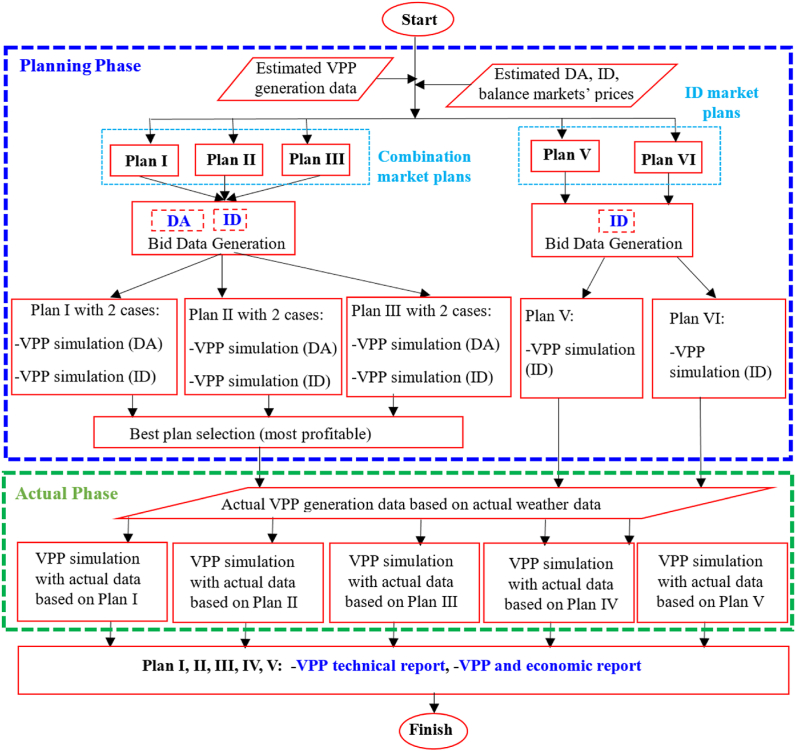
Flowchart of VPP simulation under combination and ID market plans.

For each plan detailed in Table 1, a simulation model is executed twice to generate expected and actual results, as illustrated in Fig. 2. The expected results are based on power generation forecasts during the planning phase, while the actual results are derived from real power generation data during the operational phase. During the planning phase, two scenarios are considered for bid determination. In the first scenario, if the forecasted DER generation for a specific settlement period is below the market MPR, the corresponding bid is set to zero. In the second scenario, if the forecasted DER generation is below the Internal MPR, the bid is also zero.

Every plan given in Tables 1 and is implemented for different VPP sizes. The average VPP generation size, based on the collected renewable power generation data from Tokyo Electric Power Company's website (TEPCO) [30], is 1.5 [GW]. This study scales down the VPP size to 0.05 [MW], 0.1 [MW], …, 1 [MW] to examine the profitability of small-scale VPPs that would participate in JEPX. To prevent the study acquiring proportional results, a set of random numbers is generated from uniform distribution and added into the scaled data via Equation (1).

(1) $$S_{t}^{VPP,+}=\left\{ \begin{array}{cc} S_{t}^{VPP,+}+u_{t} & if\;S_{t}^{VPP,+}+u_{t}\geq 0\\ 0 & if\;S_{t}^{VPP,+}+u_{t}<0 \end{array}\right.$$

$$u_{t}=Uniform\;distribution\;\left[-\lfloor 0.05\times VPP\;size\rfloor ,\lfloor 0.05\times VPP\;size\rfloor \right]$$

where the floor of *x* value is shown by ⌊x⌋, and “+” sign is replaced with the estimated and actual power supply of the VPP system. For each VPP size, a total of 13 simulation models are executed, consisting of 8 simulations during the planning phase and 5 simulations during the actual phase. The objective function of the VPP simulation model is designed to maximize total profit in both phases. Total profit is calculated as the difference between total income and total cost, based on the following assumptions.

Assumptions Regarding Surplus and Shortage Power Transactions: After the gate closure and dispatch, the VPP system forfeits its opportunity to buy or sell power in the ID market for the specific dispatch time. If there is a power shortage or surplus within the dispatch period, the VPP system incurs penalties from the transmission system operator.

**Fig. 3:**
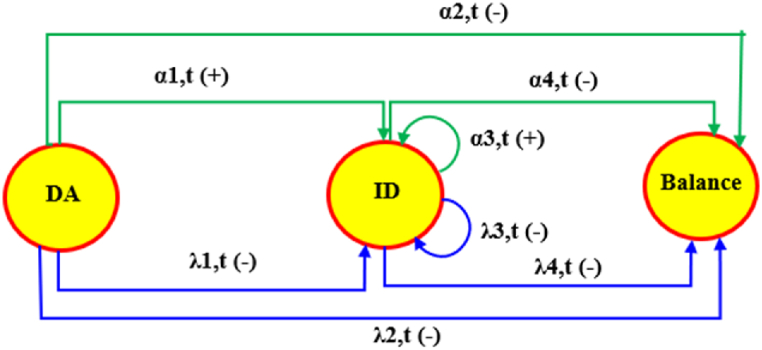
VPP market trade transition probability.

The VPP system adjusts its surplus or shortage power by either selling or buying power in the ID market at settlement period *t* with probabilities *α*_1,t_ and *λ*_1,t_, respectively (as illustrated in Fig. 3). If no adjustment is made in the ID market, the VPP system faces penalties for deviations, with the associated probabilities given in Equation (2).

(2) $$\begin{array}{c} \alpha 2,t=1-\alpha 1,t\\ \lambda 2,t=1-\lambda 1,t \end{array}$$

**Fig. 4:**
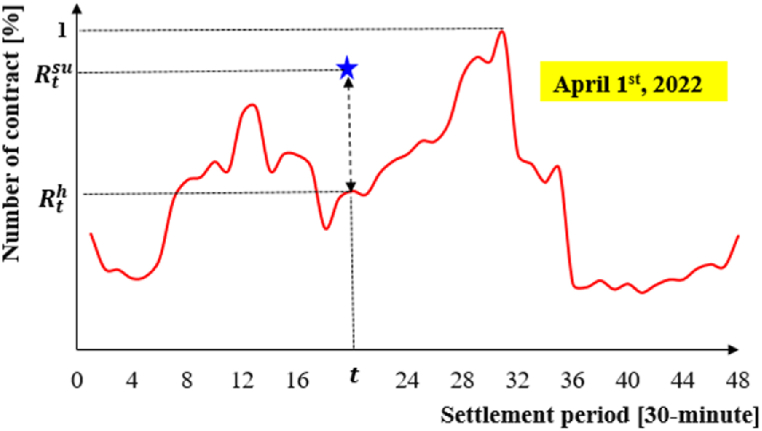
Number of contract's percentage in each settlement.

In the case of the ID market, the VPP system may adjust its bidding with probability of *α*_3,t_ and *λ*_3,t_ in the ID market. The VPP system will be penalized by the ISO system in case of any deviation with probability of *α*_4,t_ (=1- *α*_3,t_) and *λ*_4,t_ (=1- *λ*_3,t_). These probability values create a trade transition probability among three markets. The challenging issue is to find the trade transition probability values. One way to figure out the transition probability is to calculate the historical percentage of contract's number in each settlement, $R_{t}^{h}$, compared to the maximum number of daily contracts in the ID market as shown in Fig. 4. Then, for each day, this study generates 48 random numbers, $R_{t}^{su}$, from the standard uniform distribution and defines the relationships given in Equation (3).

(3) $$\left\{ \begin{array}{cc} \alpha_{1,t}=\alpha_{3,t}=\lambda_{1,t}=\lambda_{3,t}=1 & if\;R_{t}^{su}\leq R_{t}^{h}\\ \alpha_{2,t}=\alpha_{4,t}=\lambda_{2,t}=\lambda_{4,t}=0 & if\;R_{t}^{su}\leq R_{t}^{h} \end{array}\right.$$

$$\left\{ \begin{array}{cc} \alpha_{1,t}=\alpha_{3,t}=\lambda_{1,t}=\lambda_{3,t}=\frac{R_{t}^{h}}{R_{t}^{su}} & if\;R_{t}^{su}>R_{t}^{h}\\ \alpha_{2,t}=\alpha_{4,t}=\lambda_{2,t}=\lambda_{4,t}=\left(1-\frac{R_{t}^{h}}{R_{t}^{su}}\right) & if\;R_{t}^{su}>R_{t}^{h} \end{array}\right.$$

Assumption about bidding data: bidding data is managed via the minimum power generation. As shown in Table 1, a VPP system has three choices for the DA market with different MPR values. As shown in Equation (4), according to Plan I, the VPP system will select the DA market if the prediction power value is greater than or equal to 0.1 [MW] in the settlement period t. A similar condition exists for the ID market in Plan I.

(4) $$DA\;market:\left\{ \begin{array}{cc} {S_{t}^{VPP,Bid}=S}_{t}^{VPP,E} & if\;S_{t}^{VPP,E}\geq \Phi_{DA}\;\\ 0 & o.w. \end{array}\right.$$

$$ID\;market:\left\{ \begin{array}{cc} {S_{t}^{VPP,Bid}=S}_{t}^{VPP,E} & if\;S_{t}^{VPP,E}\geq \Phi_{ID}\\ 0 & o.w. \end{array}\right.$$

Equation (4) generates a single set of bidding data applicable to both the DA and ID markets, despite differing power prices at period *t* in each market. Consequently, the selection of the DA or ID market for a specific day depends on the prevailing price and the bid amount. Additionally, if the power generation falls below the minimum market requirement, the VPP cannot participate in either market. Notably, Equation (4) assumes that the shortage power is zero, as it pertains to the market MPR, not the Internal MPR. Relaxing the MPR constraint during the actual phase is expected to enhance VPP profitability. This study focuses on reducing MPR values exclusively during the planning phase (Internal MPR) for the DA and ID markets and examines how this adjustment impacts the actual VPP profit. The critical issue is determining the extent to which the Internal MPR can be reduced—i.e., the level of risk a VPP is willing to accept by submitting bids that exceed the forecasted power supply.

### 2.2 Internal minimum power requirement

The market MPR specifies the minimum power supply required from market participants. While the VPP system's capacity may meet this market MPR, its generation is subject to fluctuations due to change due to the inherent uncertainty of renewable energy sources [31]. In this context, the concept of Internal MPR refers to the minimum power supply that a VPP must maintain to mitigate the risk of significant financial losses, accounting for the uncertainties and risks associated with market decisions. Specifically, the Internal MPR represents a threshold below which the VPP's power supply may lead to incorrect market decisions, resulting in potential financial losses. To quantify the Internal MPR, the following equation is utilized:

(5) InternalMPR=Probability×Impact

The components of the right-hand side of Equation (5) are derived as follows.

- *Probability of the event occurring* (likelihood): The event computes the joint probability, as outlined in Equation (6), for the DA market, using historical data, including estimated power supply, and actual power supply.

(6) $$P\left(Estimated\;VPP\;Supply\geq \Phi_{DA,R}\cap Actual\;VPP\;supply\geq \Phi_{DA}\right)>50\%\;s.t.\Phi_{DA,R}<\Phi_{DA}$$

The value in the right-hand side of Equation (6) guarantees that in the long term, the stated risk will not lead toward the VPP's profit loss or result in an incorrect decision. In this context, an incorrect decision refers to a situation in which the VPP opts to participate in the DA market based on its estimated power supply, yet the actual power supply falls short of meeting the market MPR. Consequently, the VPP incurs a loss—either partial or total—of its profit due to having to purchase power from the ID market.

- *Impact or consequences of the event* (severity): This research utilizes historical power price data from 2016 to 2023, to derive the severity of the incorrect decision. The average power prices for the DA and ID markets over this period were 13 JPY/kWh and 12 JPY/kWh, respectively. An erroneous decision in market selection could result in a long-term loss of 1 JPY/kWh.

**Fig. 5:**
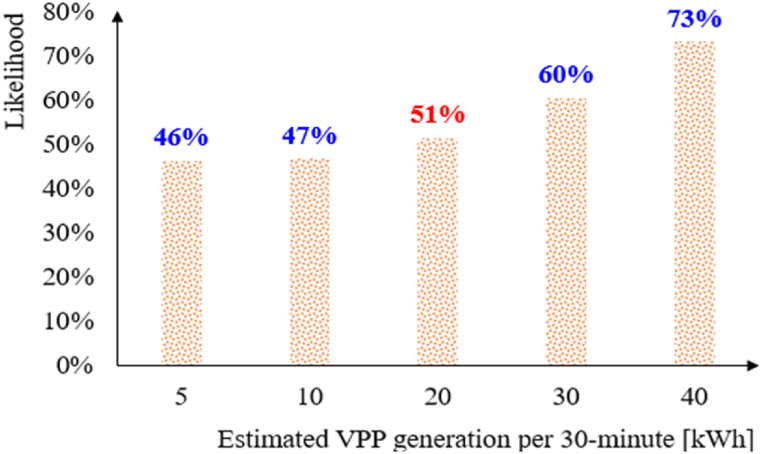
Results of the joint probability for historical data.

As illustrated in Fig. 5, the likelihood value derived from Equation (6) is 51 % for both the DA and ID markets. In other words, the Internal MPR value for both markets is 20 kWh per 30-min settlement period, equivalent to 0.04 MW ($\Phi_{DA,R}=\Phi_{ID,R}=0.04\;MW$). Consequently, the VPP system will submit its bidding data based on Equation (7) in Plans II, III, and V.

(7) $$DA\;market:\left\{ \begin{array}{ccc} {S_{t}^{VPP,Bid}=S}_{t}^{VPP,E} & & if\;S_{t}^{VPP,E}\geq \Phi_{DA}\;\\ S_{t}^{VPP,Bid}=\Phi_{DA} & & if\;{\Phi_{DA,R}\leq S}_{t}^{VPP,E}<\Phi_{DA}\\ 0 & & otherwise \end{array}\right.$$

$$ID\;market:\left\{ \begin{array}{ccc} {S_{t}^{VPP,Bid}=S}_{t}^{VPP,E} & & if\;S_{t}^{VPP,E}\geq \Phi_{ID}\\ S_{t}^{VPP,Bid}=\Phi_{ID} & & if\;{\Phi_{ID,R}\leq S}_{t}^{VPP,E}<\Phi_{ID}\\ 0 & & otherwise \end{array}\right.$$

The following pseudocode describes the algorithm's steps used in this study for all plans.

**Figure:**
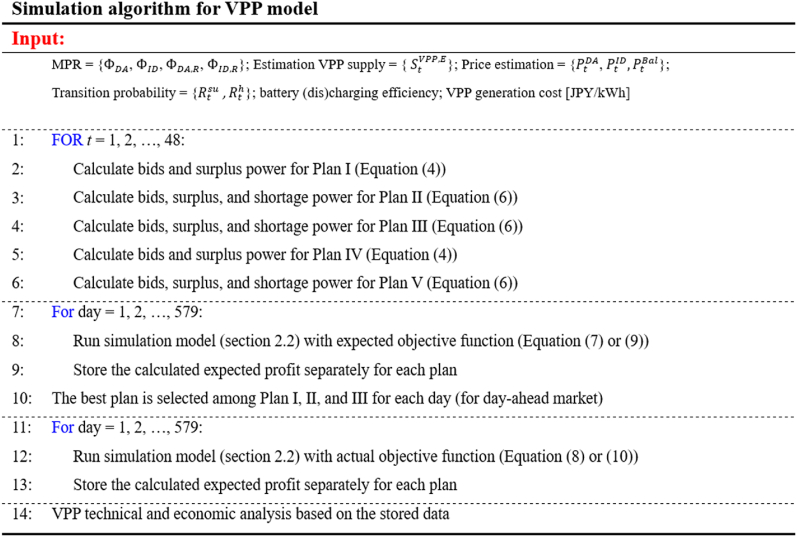

### 2.3 VPP simulation-based optimization model

The pseudocode given in the previous subsection consists of the VPP optimization model. This section explains the mathematical equations of the optimization model including the objective functions and constraints.

#### 2.3.1 VPP objective function

Equations (8), (9) are applied to calculate the expected and actual profit values in the planning and actual phases, respectively for the *combination market plans* (Plan I, II, and III).

(8) $$\mathrm{Max}\;\sum_{t=1}^{48}\left[S_{t}^{VPP,Bid}\left(B^{DA}\left[P_{t}^{DA}-S_{t}^{grid,E,ID}P_{t}^{ID}\lambda_{1,t}-S_{t}^{grid,E,Bal}P_{t}^{Bal}\lambda_{2,t}\right]+\left(1-B^{DA}\right)\left[P_{t}^{ID}-S_{t}^{grid,E,ID}P_{t}^{ID}\lambda_{3,t}-S_{t}^{grid,E,Bal}P_{t}^{Bal}\lambda_{4,t}\right]\right)-S_{t}^{VPP,E}C^{VPP}\right]$$

(9) $$\mathrm{Max}\sum_{t=1}^{48}\left[S_{t}^{VPP,Bid}\left(B^{DA}P_{t}^{DA}+\left(1-B^{DA}\right)P_{t}^{ID}\right)+{Su}_{t}^{VPP,ID}P_{t}^{ID}\alpha_{3,t}-{Su}_{t}^{VPP,Bal}P_{t}^{Bal}\alpha_{4,t}-S_{t}^{grid,A,ID}P_{t}^{ID}\lambda_{3,t}-S_{t}^{grid,A,Bal}P_{t}^{Bal}\lambda_{4,t}-S_{t}^{VPP,A}C^{VPP}\right]$$

It is notable that before running Equation (9), the binary variable, B^DA^ is determined in Equation (8). The following objective functions are used to model the VPP system under the *ID market plans* (Plan IV, and V):

(10) $$\sum_{t=1}^{48}\left[S_{t}^{VPP,Bid}P_{t}^{ID}-S_{t}^{grid,E,ID}P_{t}^{ID}\lambda_{3,t}-S_{t}^{grid,E,Bal}P_{t}^{Bal}\lambda_{4,t}-S_{t}^{VPP,E}C^{VPP}\right]$$

(11) $$\mathrm{Max}\;\sum_{t=1}^{48}\left[S_{t}^{VPP,Bid}P_{t}^{ID}+{Su}_{t}^{VPP,ID}P_{t}^{ID}\alpha_{3,t}-{Su}_{t}^{VPP,Bal}P_{t}^{Bal}\alpha_{4,t}-S_{t}^{grid,A,ID}P_{t}^{ID}\lambda_{3,t}-S_{t}^{grid,A,Bal}P_{t}^{Bal}\lambda_{4,t}-S_{t}^{VPP,A}C^{VPP}\right]$$

Equations (10), (11) indicate the objective function for the expected and actual profit values. Similar to Ref. [32], this study focuses on operation cost (running cost) of technologies to calculate the VPP profit. It is worth noting that the operation and maintenance cost of the storage was insignificant compared to the wind and solar costs ($C^{VPP}$). However, to account for the operation and maintenance costs of the battery system ($C^{Bat}$), the following Equation (12) is included in all the objective function equations previously:

(12) $$\sum_{t=1}^{48}\left[\left(S_{t}^{Bat,ch}+S_{t}^{Bat,disch}\right)\times C^{Bat,var}\right]$$

In addition to the variable operation and maintenance costs, the annual fixed operation and maintenance cost is also accounted for in the total cost, as expressed in Equation (13):

(13) $$Annual\;fixed\;operation\;cost\;of\;battery=C^{Bat,fixed}\times Battery\;Capacity\times Simulation\;period$$

#### 2.3.2 VPP constraints

The “+” sign in Equations (14), (15), (16), (17) is replaced with “A” or “E” for the actual and estimated purposes, respectively. The amount of demand is equal to the submitted bid at period *t*, and the demand load data has been replaced with VPP bid data in Equation (14). The other equations determine the amount of grid and VPP power supply along with the battery charging/discharging power.

A. *Supply-Demand balancing constraint*:

(14) $$S_{t}^{VPP,+}-{Sl}_{t}^{Negative}+{Sl}_{t}^{Positive}=S_{t}^{VPP,Bid}$$

(15) $${Sl}_{t}^{Negative}\times {Sl}_{t}^{Positive}\leq 0$$

(16) $$\frac{S_{t}^{Bat,ch}}{\eta_{ch}^{Bat}}+{Su}_{t}^{VPP}\leq {Sl}_{t}^{Negative}$$

(17) $$\eta_{disch}^{Bat}\times S_{t}^{Bat,disch}+\left(S_{t}^{grid,+,ID}+S_{t}^{grid,+,Bal}\right)\leq {Sl}_{t}^{Positive}$$

The balance between demand and supply is guaranteed via Equations (14), (15), (16), (17). The slack variables are used to adjust the amount of grid power supply and battery charging/discharging power.

B. *Technological constraints of DERs*:

The study has access to the total estimated and actual power supply of DERs (without access to details of each DER generation). Therefore, the DERs technologies’ constraints are skipped.

C. *Energy storage constraints*:

(18) $${\eta_{ch}^{Bat}\times S}_{t}^{Bat,ch}\leq Battery\;capacity$$

(19) $$\frac{S_{t}^{Bat,disch}}{\eta_{disch}^{Bat}}\leq S_{\max}^{Bat,disch}$$

(20) $$\frac{S_{t}^{Bat,disch}}{\eta_{disch}^{Bat}}\leq {SOC}_{t-1}^{Bat}$$

(21) $$\frac{S_{t}^{Bat,disch}}{\eta_{disch}^{Bat}}\leq {Sl}_{t}^{Positive}$$

(22) $$\frac{S_{t}^{Bat,disch}}{\eta_{disch}^{Bat}}\geq {SOC}_{t-1}^{Bat}-M_{1}\times \left(1-B_{t}^{Bat,disch}\right)$$

(23) $$\frac{S_{t}^{Bat,disch}}{\eta_{disch}^{Bat}}\geq {Sl}_{t}^{Positive}-M_{1}\times B_{t}^{Bat,disch}$$

(24) $${\eta_{ch}^{Bat}\times S}_{t}^{Bat,ch}\leq Battery\;capacity-{SOC}_{t-1}^{Bat}$$

(25) $${\eta_{ch}^{Bat}\times S}_{t}^{Bat,ch}\leq {Sl}_{t}^{Negative}$$

(26) $${\eta_{ch}^{Bat}\times S}_{t}^{Bat,ch}\geq \left(Battery\;capacity-{SOC}_{t-1}^{Bat}\right)-M_{1}\times \left(1-B_{t}^{Bat,ch}\right)$$

(27) $${\eta_{ch}^{Bat}\times S}_{t}^{Bat,ch}\geq {Sl}_{t}^{Negative}-M_{1}\times B_{t}^{Bat,ch}$$

(28) $${SOC}_{t}^{Bat}={SOC}_{t-1}^{Bat}-\frac{S_{t}^{Bat,disch}}{\eta_{disch}^{Bat}}+{\eta_{ch}^{Bat}\times S}_{t}^{Bat,ch}$$

(29) $${SOC}^{\min}\leq {SOC}_{t}^{Bat}\leq {SOC}^{\max}$$

(30) $${SOC}_{t=0}^{Bat}=Initial\;SOC$$

Equations (18), (19) set the upper bounds for charging and discharging power of battery in each period. Equations (20), (27) confine the amount of discharging and charging power from/into the battery. Equations (28), (29) represent the energy balance of the battery and its boundaries. Equation (30) finally assigns an initial value for the battery's state of charge. Equations (31), (32), (33) are used to calculate the battery capacity for each VPP system:

(31) $$Battery\;capacity\;\left[Ah\right]=\frac{Daily\;power\;load\;\left[Wh\right]\times autonomoy\;day\times temperature\;correction\;factor}{Battery\;voltage\times Load\;subsystem\;efficiency\times DoD}$$

where the daily power load is calculated as follows:

(32) $$\mathrm{Max}\;daily\;power\;load\;\left[Wh\right]=\max\left\{S_{t}^{VPP,A}-S_{t}^{VPP,Bid}\right\}\;t=1,\ldots ,48$$

(33) $$Daily\;power\;load\;\left[Wh\right]=\frac{\sum_{d=1}^{579}\mathrm{Max}\;daily\;power\;load\;\left[Wh\right]}{579}$$

### 2.4 Model's data

Renewable energy data, including wind and solar power data, was obtained from TEPCO [30]. Moreover, demand load data and non-renewable energy data were also collected from TEPCO, just to have an idea of the bid submission's data. As shown in Table 2, the scope of data is seven years (2016–2023) with 30-min resolution. Electric power price in the DA and ID markets along with number of power trading contracts in the ID market were gathered from the JEPX website [28]. The scope and data resolution were the same as power data. The imbalance cost data with resolution of 30-min was obtained from the imbalance cost calculation service website [33] for Tokyo Metropolis area. The scope of the imbalance cost was shorter than the previous data and it included April 2022 to October 2023. Thus, the simulation models were implemented for all data from April 2022 until October 2023.

**Table 2:** Data types and their sources.

Data	Type of data	Data collected period	Data resolution	Reference
Demand load	Estimated	2016/04/01–2023/10/31	30-min	[30]
	Actual			
Renewable power generation	Estimated			
	Actual			
Non-renewable power generation	Estimated			
	Actual			
Electric power price	Day-ahead	2016/04/01–2023/10/31	30-min	[28]
	Intra-day			
Number of power trading contracts	Intra-day			
Electric imbalance cost	Actual	2022/04/01–2023/10/31	30-min	[33]

## 3 Results

Prior to presenting the simulation results, the key assumptions and parameters employed in this study are outlined to ensure greater transparency.

### 3.1 Assumptions and parameters used in the simulation models

VPP capacity and its power generation variability: This study investigates five plans across 11 VPPs during both the planning and actual phases, as detailed in Table 1. As illustrated in Fig. 2, the planning phase comprises eight simulation cases, while the actual phase includes five, resulting in a total of 13 simulation cases. For each VPP size, these 13 simulation cases are executed. The VPP capacities, as presented in Table 3, are allocated across all plans after being influenced by random factors described in Equation (1).

**Table 3:** Initial values of the optimization model's parameters.

VPP size [MW]		0.05	0.1	0.2	0.3	0.4	0.5	0.6	0.7	0.8	0.9	1
Battery bank capacity [kWh]		20	40	79	119	158	197	237	276	315	354	394
**Estimated VPP generation [kWh/30-min]**	**min**	**0**	**0**	**0**	**0**	**0**	**0**	**0**	**0**	**0**	**0**	**0**
	**Ave.**	**25**	**50**	**99**	**149**	**199**	**249**	**298**	**348**	**398**	**447**	**497**
	**Max**	**134**	**271**	**537**	**804**	**1068**	**1341**	**1609**	**1870**	**2157**	**2420**	**2674**
**Actual VPP generation [kWh/30-min]**	**min**	**0**	**0**	**0**	**0**	**0**	**0**	**0**	**0**	**0**	**0**	**0**
	**Ave.**	**25**	**50**	**100**	**150**	**200**	**250**	**300**	**350**	**400**	**450**	**500**
	**Max**	**140**	**282**	**565**	**848**	**1134**	**1415**	**1678**	**1942**	**2246**	**2530**	**2838**

**Fig. 6:**
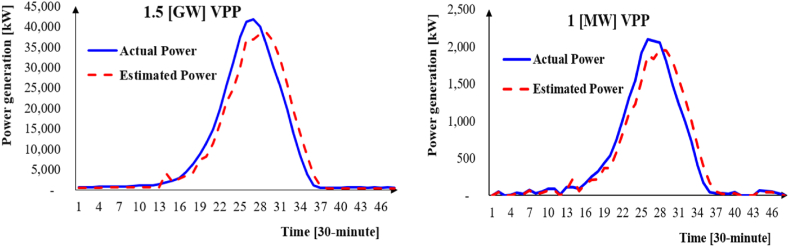
Actual (left) and scaled-down (right) VPP power generation on January 4, 2022.

As shown in Fig. 6, these random factors introduce slight variations in power generation for the actual 1.5 MW VPP, preventing proportional results from being obtained. Table 3 represents the VPP size, storage capacity, and descriptive statistics of the actual and estimated VPP power supply from April 2022 to October 2023. It is assumed that the battery SOC is full at the initial time. The study calculates the absolute value of the difference between actual and estimated VPP power generation for each settlement period, *t*. Equation (32) is applied for the absolute values to calculate the maximum daily power load. The average metric in Equation (33) smooths the outliers among daily power load data.

Battery capacity and its parameters: The battery capacity for each VPP is calculated using Equation (31), based on parameters and specifications provided in Table 4 [34].

**Table 4:** Battery capacity calculation data.

Initial state of charge [%]	100	Day of autonomy [day]	1
Max state of charge [%]	80	Voltage [V]	24
Min state of charge [%]	20	Load subsystem efficiency [%]	85
Charging/Discharging efficiency [%]	90	Depth of discharge, DoD [%]	95
Temperature compensation factor	1.19	Simulation period [year]	1.58

Cost assumptions: Historical cost data for the DA and ID markets are incorporated into the simulation models. VPP supply cost is calculated from the operation and maintenance cost of wind turbine [35,36] and solar power [[37], [38], [39]] which are 4.5 [JPY/kWh] and 2.45 [JPY/kWh], respectively. The shares of solar and wind power from Japan's total electricity generation were 9.9 % and 0.9 %, respectively in 2022 [40]. Therefore, the estimated operation and maintenance cost of the VPP system in this study is set to 2.6208 JPY/kWh.^6^ In the case of battery, its operation and maintenance cost (variable cost) is estimated 0.3 cents/kWh/year, while its fixed value ranges between $6–20/kW/year [41]. The average value ($13/kW/year) is considered as a fixed cost of battery in this study.

### 3.2 VPP simulation models’ results

A total of 143 simulation models were performed for 11 VPP sizes (13 simulation models for each size). The results were divided into technical and economic parts.

#### 3.2.1 VPP technical results

**Fig. 7:**
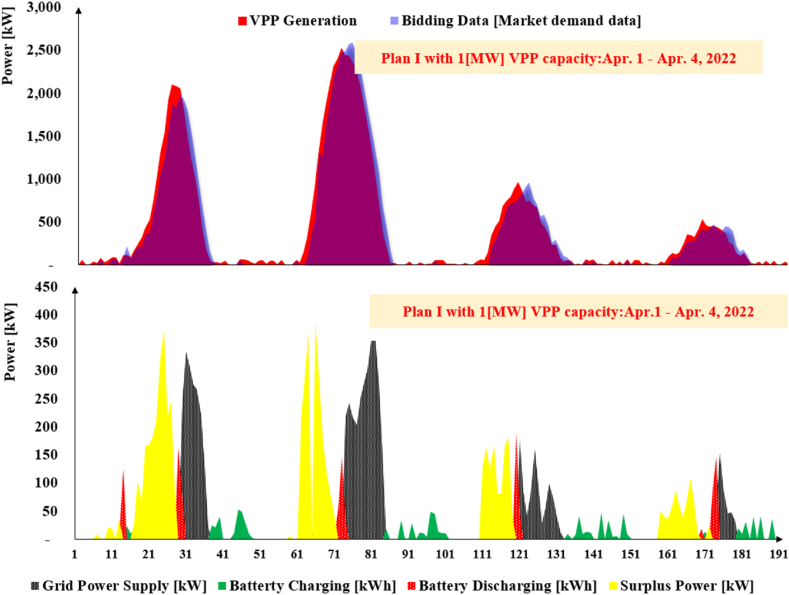
The upper half of the graph represents the bid and actual VPP generation data under Plan I. The lower half of the graph indicates power supply by grid, battery charge and discharge as well as surplus power.

Fig. 7 shows the results of the VPP simulation model under Plan I with 1[MW] capacity for four consecutive days (Apr.1–4, 2022). The upper graph displays the demand (submitted bid) and VPP actual power supply, while the lower graph demonstrates the share of VPP, energy storage and grid power supply in 192 settlement periods. The gap between the VPP generation and bidding data implies the forecasting inaccuracy. There are various settlement periods in which the VPP system generates power, but does not meet the market MPR. For these periods, the VPP has not submitted any bidding data.

The yellow part in the lower graph represents the amount of surplus power generated by the VPP system which either sells in the ID market or receives penalty from the ISO. Grid power supply in the lower graph indicates the periods in which the VPP system has generated lower power than the submitted bidding data.

##### 3.2.1.1 VPP technical results under combination market plans

**Fig. 8:**
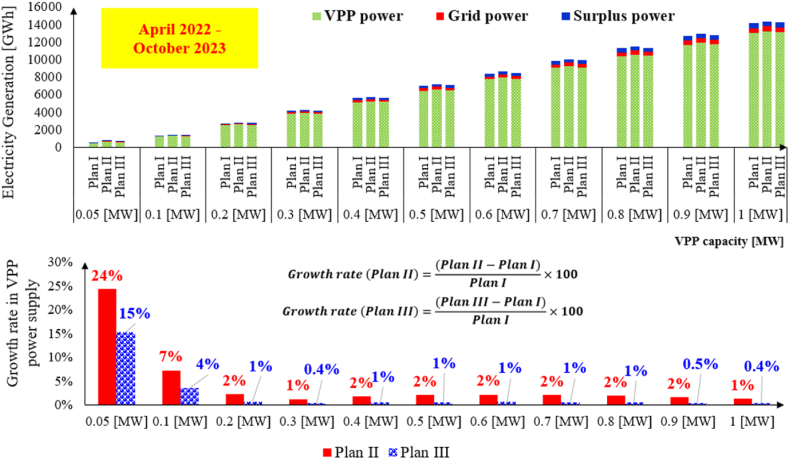
Power generation under combination market plans.

Fig. 8 shows electricity generation under combination market plans. Compared to Plan II, two other plans have supplied more power into the market. The lower graph indicates that the growth rate of power supply in Plan II is more than Plan III because of the Internal MPR effect. The Internal MPR reduces the market MPR and causes more small-scale VPP engagement into the power market. The effect of the Internal MPR is significant when the size of VPP system decreases. For example, the growth rates of VPP power supply with capacity of 0.05 [MW] are 24 % and 15 %.

**Fig. 9:**
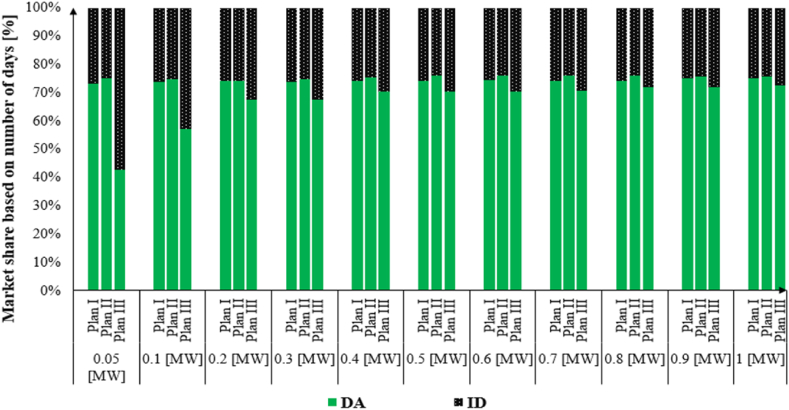
Market share under combination market plans.

Fig. 9 represents the market share of Plan I, Plan II and Plan III based on the number of days. The dominant market for all VPPs greater than 0.05 [MW] is the DA market. The share of the DA market varies between 73 % and 75%in Plan I. While the share of the DA market in Plan II is slightly more than Plan I (74 %–76 %). In contrast, the share of the DA market for Plan III changes from 43 % to 73 % for VPP capacity with 0.05 [MW] to 1 [MW], respectively. In the case of Plan III with VPP capacity of 0.05 [MW], the Internal MPR affects just for the ID market. Thus, the VPP system submit a bid for various settlement periods which were unable to submit a bid due to the market MPR.

##### 3.2.1.2 VPP technical results under ID market plans

**Fig. 10:**
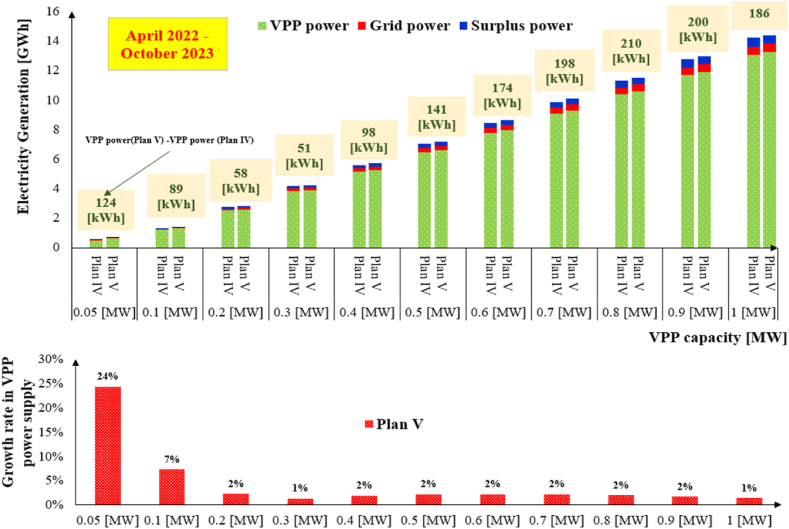
Power generation under the ID market plans.

Fig. 10 shows electric power generation in terms of VPP, grid, and surplus power under Plan IV and Plan V.

The difference between two plans given in the upper side of the figure represents that applying the Internal MPR increases the VPP power supply in the ID market. The lower part of Fig. 10 shows the growth rate of the VPP power supply in Plan V due to the Internal MPR. The VPP system in Plan V with 0.05 [MW] capacity supplies power up to 24 % more than Plan IV (equivalent to 124 [kWh]). The additional power supply in Plan V is just because of the Internal MPR.

#### 3.2.2 VPP economic results

This section presents the economic results of the research in terms of combined and single ID market plans.

##### 3.2.2.1 VPP economic results under combination market plans

**Fig. 11:**
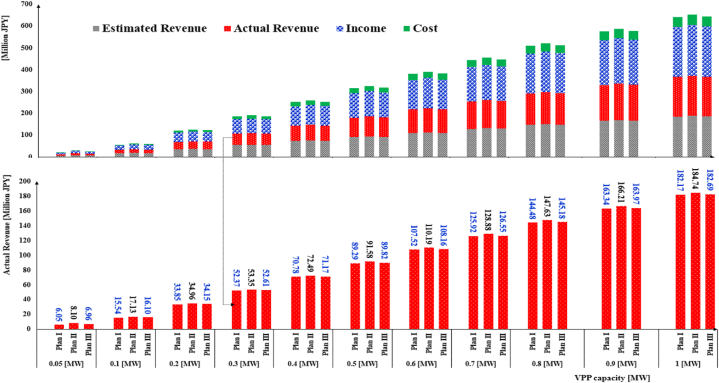
Economic metrics under combination market plans.

Fig. 11 displays the cost analysis of the combination market plans in terms of the estimated and actual profit values. The share of income and cost for actual plans is also given. According to the lower graph, actual profit under Plan II in all VPP sizes is bigger than Plan I and Plan III. This finding again highlights the need to change in JEPX minimum power requirement. According to the finding, the Internal MPR mechanism provides more profit for the small-scale VPP system. This mechanism can be figured out by the JEPX committee or adopted by the VPP owners within bid submission. For instance, in the case of 0.05 [MW] capacity, the VPP earns 2.05 × 10^6^ [JPY] more profit than Plan I within 579 days.

##### 3.2.2.2 VPP economic results under ID market plans

**Fig. 12:**
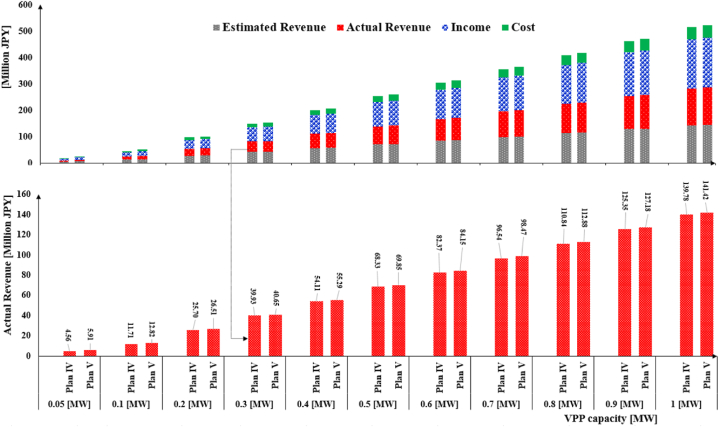
Economic metrics under ID market plans.

Fig. 12 demonstrates the economic metrics under the ID market plans. The main difference between Plan IV and Plan V is the Internal MPR because both plans have been simulated in the ID market.

The effect of the Internal MPR is evident for small VPP sizes. For example, by replacing the market MPR with the Internal MPR, the VPP profit increases up to 1.35 × 10^6^ [JPY] in the VPP with 0.05 [MW] capacity. Although the lower graph illustrates a slight increase in actual revenue in Plan V, the gap between two plans’ revenues narrows down by growing the VPP size. It implies that a VPP system with a bigger capacity mostly generates power more than 0.1 [MW].

**Fig. 13:**
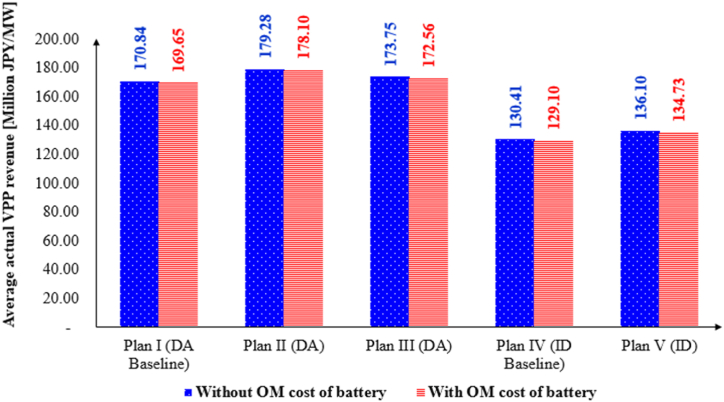
Plan's rank in terms of average actual profit.

Fig. 13 incorporates the baseline conditions for the DA and ID markets, as well as various market strategies (Plans II, III, and V) over a period of 19 months (1.58 years). It illustrates the average actual profit per megawatt for each strategy, both with and without the inclusion of battery operation and maintenance costs. The inclusion of the battery operation costs results in a slight reduction in the VPP profit. To compute the average actual profit per megawatt, the actual profit of each plan was normalized by its capacity, and then the average profit across 11 VPPs was calculated. According to Fig. 13, Plan II achieves the highest average profit among all plans, followed by Plan III and Plan I within the context of the combined market plans. In the ID market, Plan V generates more revenue compared to Plan IV. These findings underscore the potential benefits of modifying the market MPR to increase opportunities for small-scale VPP systems to participate in the DA market.

### 3.3 Internal MPR effect on JEPX power market and grid reliability

Although Fig. 13 illustrates the potential changes in the profitability of all VPP systems over one year under the Internal MPR, it does not account for the risks associated with JEPX price fluctuations or grid reliability. Incorporating these factors is crucial for providing a more comprehensive understanding of the Internal MPR, offering valuable insights for decision-makers.

**Fig. 14:**
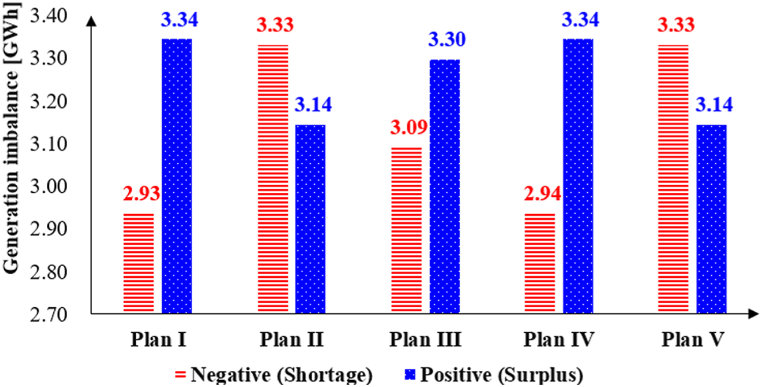
Power generation imbalance for all VPPs.

*Grid reliability*: To assess grid reliability under the Internal MPR, detailed data is required which is not available. For simplicity, this study evaluates power generation imbalance, as a metric, across all VPPs to compare the market strategies (Plans II, III, and V) with current JEPX market plans (Plans I and IV). Fig. 14 presents the positive and negative power generation imbalances for all VPPs across the five plans. Compared to Plan I, the negative generation imbalance increases in Plans II and III, with a similar trend observed for Plan V relative to Plan IV. These results suggest that the power grid must have sufficient flexibility to address shortages in VPP generation. In the case of positive generation imbalances (surplus), a corresponding pattern is observed in the reverse way, indicating the need for the VPP system to manage the surplus. If unmanaged, the system operator must intervene to balance excess electricity through storage, redirection, or curtailment to maintain grid stability.

**Fig. 15:**
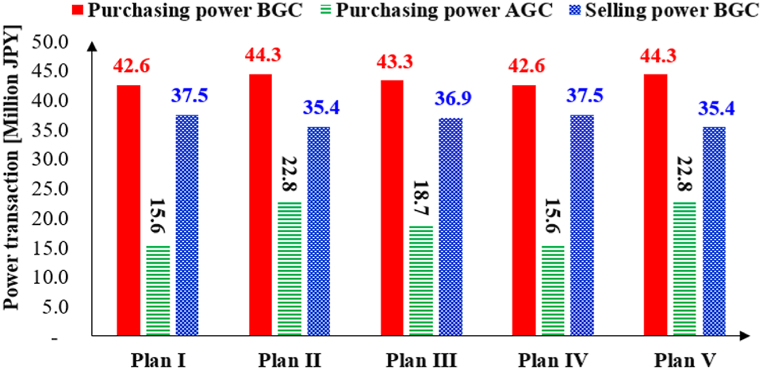
Power transaction Before and After Gate Closure (BGC, AGC) all VPPs.

*JEPX Power market*: Although the Internal MPR may increase the electricity supply in the market, potentially lowering power prices, it could also introduce greater price variability if small-scale VPPs, with their less predictable generation profiles, fail to consistently match demand. As shown in Fig. 15, the volume of purchasing power in Plans II and III exceeds that in Plan I, particularly in the purchasing power after gate closure (AGC). Given the limited time to purchase shortage power after gate closure, this is likely to drive up the price per unit of selling power. A similar trend is observed for purchasing power in Plan V compared to Plan IV. In contrast, the selling power before gate closure (BGC) in Plans II, III, and V has decreased relative to their corresponding base-case plans (Plans I and IV). This reduction in power transactions creates an imbalance between supply and demand in the ID market, which is likely to increase power prices in the ID market.

Thus, although the Internal MPR could lead to increased participation of small-scale VPPs in the JEPX market and their profits, it could also increase market volatility due to the greater number of smaller, more variable entities participating in the market. The effect on market and grid stability would depend on how well these smaller VPPs can forecast and manage power generation, as well as the ability of market and grid operators to balance supply and demand effectively.

*Possible Changes in long-term VPP profitability*: As shown in Fig. 15, the purchasing power before and after gate closure increases by 4 % and 46 %, respectively, for Plan II when compared to the existing combination plan in the JEPX market (Plan I). For Plan III, the corresponding increases are 2 % and 20 %. Similarly, for Plan V, the purchasing power before and after gate closure increases by 4 % and 46 %, respectively, compared to the existing ID plan in the JEPX market (Plan IV). In contrast, the selling power in the ID market decreases across all plans under the Internal MPR option. Consequently, it is expected that the introduction of the Internal MPR will drive an increase in power prices in both the ID (before gate closure) and balancing (after gate closure) markets.

**Fig. 16:**
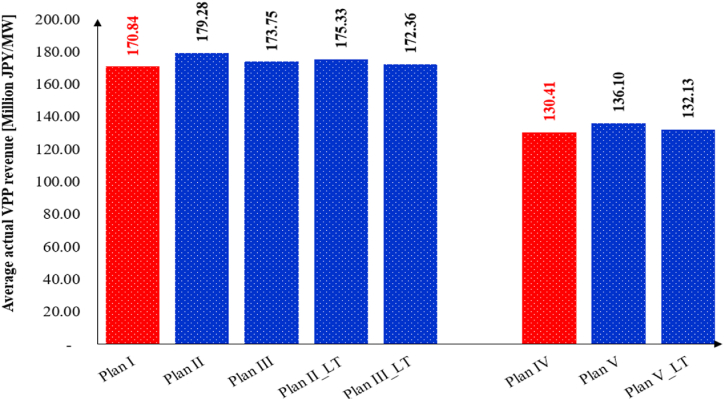
Possible changes in long-term VPP profitability (without operation cost of battery).

This study assumes that the price variations in the ID and balancing markets remain consistent with the changes in purchasing power before and after gate closure in the event of any power shortage for the VPP system. Based on this assumption, potential long-term changes in VPP profitability are evaluated for Plans II, III, and V (designated as Plan II_LT, Plan III_LT, and Plan V_LT). Fig. 16 illustrates that the price changes induced by the introduction of the Internal MPR lead to a reduction in the average actual VPP profit for Plan II_LT, Plan III_LT, and Plan V_LT. Although the price changes result in a reduction in VPP profit—such as a decrease from 179.28 million JPY/MW to 175.33 million JPY/MW in Plan II—the VPP system under the Internal MPR still achieves higher profits compared to the baseline plans.

## 4 Discussion

This study utilized historical data^7^ and the joint probability equation to determine the Internal MPR, which was found to be 0.04 MW. The introduction of this Internal MPR allows small-scale VPP systems to participate more effectively in the DA and ID markets, thereby increasing potential profits. The comparison between Plan I and Plan II, as well as Plan IV and Plan V (see Fig. 13), highlights that VPP profits improved with the implementation of the Internal MPR.

*Suggestion for JEPX market*: To encourage active participation by small-scale VPP systems in the DA and ID markets, it is suggested that the JEPX consider relaxing the market MPR. A reduction in the market MPR would not only enhance the profitability for small-scale VPPs but also increase the proportion of renewable energy in the overall power mix. Conversely, VPP owners could use the method outlined in this paper to calculate their own Internal MPR values, potentially leading to increased profits. The results indicate that, even with potential price increases in the ID and balancing markets, the average actual VPP profit under the Internal MPR is higher than that of the existing plans in the JEPX market. While the DA and ID markets offer greater profit opportunities for small-scale VPPs, the balancing market imposes more stringent requirements, including a minimum bid of 1 MW for durations ranging from 5 min to 4 h [42].

*Applicability to Other Markets*: Power markets in Europe are exploring innovative solutions, such as hybrid power generation models within the framework of VPPs, to enhance the integration of renewable energy sources into national energy mixes [43]. The minimum power requirement for the DA market within the European Power Exchange (EPEX) is set at 0.1 MW, which is consistent with the minimum quantity requirement in the JEPX [44]. The application of the proposed Internal MPR could provide valuable insights into the potential effects of reduced MPRs on market dynamics, particularly in terms of fostering an increased share of renewable energy in the power generation portfolio of these countries.

## 5 Conclusion

This study analyzed the existing day-ahead (DA) and intraday (ID) Japan Electric Power eXchange (JEPX) market based on the internal minimum power requirement (MPR). The MPR value for the DA and ID markets is 0.1 [MW] for every 30-min settlement period. This study designed three plans (Plan II, Plan III, and Plan V) to evaluate the effects of the Internal MPR on profitability of 11 small-scale VPP systems. The Internal MPR set 0.04 [MW] capacity for bid submission in the DA and ID markets in Plan II. The Internal MPR set 0.04 [MW] just for the ID market in Plan III and Plan V. The stated plans were compared with the existing plans in the JEPX (Plan I and Plan IV).

The results indicate that implementing the Internal MPR led to increased profits for all 11 VPPs across Plans II, III, and V. Plan II yielded the highest profit, followed by Plan III and Plan I under the combined market plans. Additionally, Plan V achieved greater profits than Plan IV in the ID market. Specifically, the average actual profit per megawatt increased from 170.84 million Japanese yen (JPY) in Plan I to 179.28 million JPY in Plan II. For a VPP with a 0.05 MW capacity, Plan II generated an additional 40.95 × 10^6^ JPY in profit compared to Plan I in the combined market. In the ID market, Plan V provided 27.07 × 10^6^ JPY more profit than Plan IV for the same capacity.

These findings suggest that reducing the market MPR in the DA and ID markets could enhance small-scale VPP engagement and profitability, aligning with the government's carbon-neutral goals for 2050. It is important to note that the calculated profits consider interactions among the DA and ID markets and the probabilistic nature of imbalance settlements for surplus and shortage power. Although the objective function of the model was to maximize VPP profitability, priority was given to utilizing renewable power over less expensive fossil fuel power in certain time slots. Future research will explore the impact of more accurate forecasting models on VPP profitability, incorporating statistical and deep learning methods to improve the prediction of VPP power generation in the DA market.

## CRediT authorship contribution statement

**Reza Nadimi:** Writing – review & editing, Writing – original draft, Visualization, Validation, Methodology, Investigation, Formal analysis, Data curation, Conceptualization. **Masahito Takahashi:** Writing – review & editing, Validation, Conceptualization. **Koji Tokimatsu:** Writing – review & editing, Validation, Methodology, Conceptualization. **Mika Goto:** Writing – review & editing, Validation, Supervision, Resources, Project administration, Methodology, Funding acquisition, Conceptualization.

## Data availability statement

The dataset created for this study can be accessed in the following GitHub repository.: https://github.com/Nadimi-Reza/VPP-Japan-Power-Market.git.

## Funding

This work was supported by Council for Science, Technology, and Innovation (CSTI), Cross-ministerial Strategic Innovation Promotion Program (SIP), the 3rd period of SIP “Smart energy management system” Grant Number JPJ012207 (Funding agency: JST).

## Declaration of competing interest

The authors declare that they have no known competing financial interests or personal relationships that could have appeared to influence the work reported in this paper.

## Footnotes

1 Accepted maximum duration with faults present before starting corrective maintenance.

2 The dispatch interval and the settlement period differ in some power markets.

3 VPPs can enhance their profitability by leveraging energy storage systems to buy and sell power at different price points, while also maximizing the charge and discharge cycles of the storage systems.

4 Multi-interval one-shot method or multi-interval rolling-window method.

5 [Internal MPR, Market MPR].

6 2.6208 = 0.067 × 145.61 (As of December 13, 2023, the exchange rate is as 1 USD = 145.61 JPY).

7 Power prices, estimation and actual renewable energy generation.
